# A reflection on ‘A hydrazone-based covalent organic framework for photocatalytic hydrogen production’: teaching sponges new tricks

**DOI:** 10.1039/d6sc90032a

**Published:** 2026-03-11

**Authors:** Andrés Rodríguez-Camargo, Bettina V. Lotsch

**Affiliations:** a Nanochemistry Department, Max Planck Institute for Solid State Research 70569 Stuttgart Germany b.lotsch@fkf.mpg.de; b Department of Chemistry, University of Stuttgart 70569 Stuttgart Germany; c Department of Chemistry, University of Munich (LMU) 81377 Munich Germany

## Abstract

Covalent organic frameworks (COFs) are a unique class of porous materials built entirely from organic building blocks. As such, COFs unite the tunability of molecules with the robustness and optoelectronic functionality of extended solids—key requisites for (photo)catalysis. This LEGO®-like design of crystalline “molecular sponges” has captivated the imagination of chemists and inspired the first COF photocatalyst: a hydrazone-linked COF capable of harnessing visible light to drive the evolution of hydrogen from water. This commentary revisits that seminal contribution, published 11 years ago in *Chemical Science* (L. Stegbauer, K. Schwinghammer, B. V. Lotsch, *Chem. Sci.*, 2014, **5**, 2789–2793, https://doi.org/10.1039/C4SC00016A), and reflects on its lasting impact. We survey the major advances that have shaped COF photocatalysis over the past decade and outline emerging opportunities and challenges, offering a forward-looking perspective on the role of COFs in solar energy conversion.

## Abstract 2

In celebration of our 15th anniversary and some of our most popular articles, Bettina Lotsch reflects on the area of catalysis with covalent organic frameworks, building on a seminal paper published in *Chemical Science* on this topic (*Chem. Sci.*, 2014, https://doi.org/10.1039/C4SC00016A).

## Graphical abstract

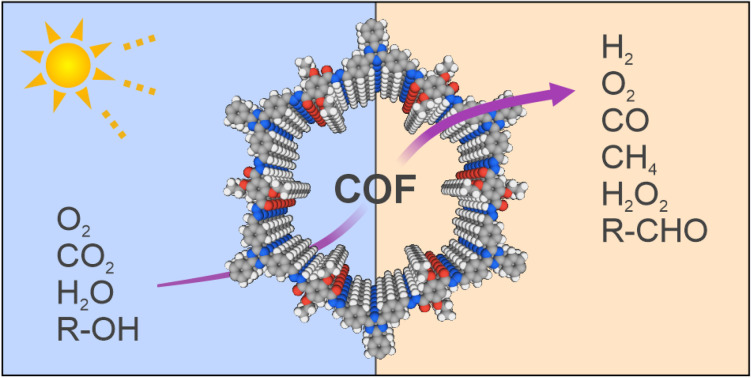

## Introduction

The rapid increase in atmospheric CO_2_ levels urges the development of new strategies to meet global energy needs using renewable sources. Solar fuels in which solar energy is stored in the form of chemical bonds, inspired by natural photosynthesis, represent an attractive building block in the ongoing energy transition. The most direct approach to harness solar energy is to convert sunlight into valuable “green” fuels (*e.g.*, hydrogen, methanol). In this context, photocatalytic water splitting, where hydrogen and oxygen are generated under light irradiation in the presence of a catalyst (or a combination of catalysts) stands out as a primary route. Developing both efficient and stable catalysts is essential to bring solar fuel production to a techno-economically viable and industrially relevant level. A groundbreaking milestone was achieved by Fujishima and Honda in 1972, who demonstrated the photogeneration of hydrogen using TiO_2_ under light irradiation—marking the beginning of solar fuel photocatalysis.^1^ In the following decades, significant progress was made with inorganic photocatalysts, while the introduction of “soft” organic photocatalysts such as carbon nitrides expanded the field, offering promising activity but limited tunability.^2,3^ The discovery of a new generation of photocatalysts based on COFs, which can be precision-made by molecular design, therefore marks a milestone in “soft photocatalysis”.

COFs are crystalline porous solids constructed entirely from organic building blocks. Their purely organic nature endows them with the vast structural diversity and tunability of organic chemistry, where both molecular design and post-synthetic engineering expand the palette of possible architectures and functionalities. Typically, their building units—often referred to as linkers—are aromatic systems that can enable extended π-conjugation throughout the framework, thereby imparting semiconducting electronic and optical properties. Exploiting these features, in 2014 we reported the first example of COFs employed as photocatalysts for solar fuel production. In that study, a hydrazone-based COF, TFPT–COF, was applied to the photocatalytic hydrogen evolution reaction (HER). This seminal work demonstrated the remarkable potential of COFs as photocatalysts and paved the way for extensive research into their application in solar fuel generation over the following years (https://doi.org/10.1039/C4SC00016A).^4^

### A decade of progress

**Fig. 1:**
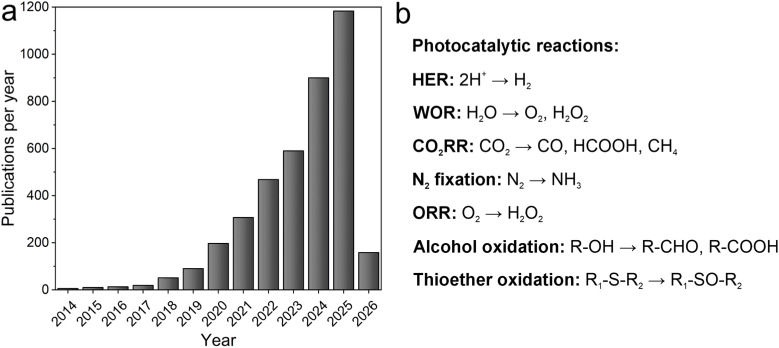
(a) Number of publications per year registered in SciFinder with the keywords (“Covalent organic framework” OR “Covalent triazine framework” AND photocat*). Data retrieved on January 27th, 2026. (b) Key photocatalytic reactions in which COFs have been utilized as photocatalysts.

Our 2014 *Chem. Sci.* publication catalyzed the exploration of COFs as photocatalysts, sparking an exponential rise in research activity in this field. Within a decade, the number of related publications surpassed a thousand per year (Fig. 1a). This rapid growth highlights the promising role of COFs in photocatalytic applications and underscores the strong attention they have attracted within the scientific community. In the early stages, the HER was at the spotlight of COF-based photocatalysis. COFs acted as light-absorbing materials (*i.e.*, photosensitizers) and, when combined with a co-catalyst—typically platinum nanoparticles—and a sacrificial electron donor, enabled continuous H_2_ generation under visible light. Over the past decade, the concerted efforts of researchers have significantly advanced this reaction, making it feasible under more sustainable conditions. Notably, the reliance on noble-metal co-catalysts such as Pt has increasingly been supplanted by earth-abundant alternatives such as Ni,^5^ and the use of sacrificial electron donors has been eliminated by coupling the HER with the oxygen evolution reaction (OER) in an overall water-splitting process.^6^

Building on their excellent performance in the HER, COF-based systems have been extended to other photocatalytic reactions, including the CO_2_ reduction reaction (CO_2_RR)—where carbon monoxide, formic acid, and methane are the most commonly reported photoreduction products—as well as nitrogen fixation for photocatalytic ammonia generation, and hydrogen peroxide (H_2_O_2_) production *via* the oxygen reduction reaction (ORR) and water oxidation reaction (WOR, Fig. 1b).^7–12^ Among these, H_2_O_2_ production has attracted particular attention following its first report in 2020.^13^ The direct utilization of oxygen, water, and natural sunlight as feedstocks renders the photocatalytic synthesis of H_2_O_2_ a particularly appealing route for solar-to-chemical fuel conversion. Furthermore, the intrinsic structural tunability and chemical versatility of COFs have enabled the design of highly efficient photocatalysts, achieving remarkable metrics such as an H_2_O_2_ production rate of 7.2 mmol g^−1^ h^−1^, an apparent quantum yield of 18.0%, and a solar-to-chemical efficiency of 0.91%, using only water, air, and light.^14^ Beyond their activity, the versatility of COF photocatalysts also facilitates their integration into practically relevant configurations, including flow reactors and film-based architectures, which are promising for future scalable photocatalytic applications.

As mentioned above, COFs can efficiently activate molecular oxygen under irradiation with visible light, which not only enables H_2_O_2_ production *via* the ORR but also provides a versatile platform for aerobic oxidation reactions. Consequently, numerous studies have coupled oxygen reduction with the oxidation of alcohols and thioethers to photogenerate value-added organic molecules (Fig. 1b). The exploration of these reactions positions COFs as photocatalysts beyond solar fuel production, opening opportunities for the sustainable synthesis of fine chemicals through photoredox catalysis, thereby significantly broadening the application scope of COFs in catalysis.

### Decoding the photocatalytic power of COFs

**Fig. 2:**
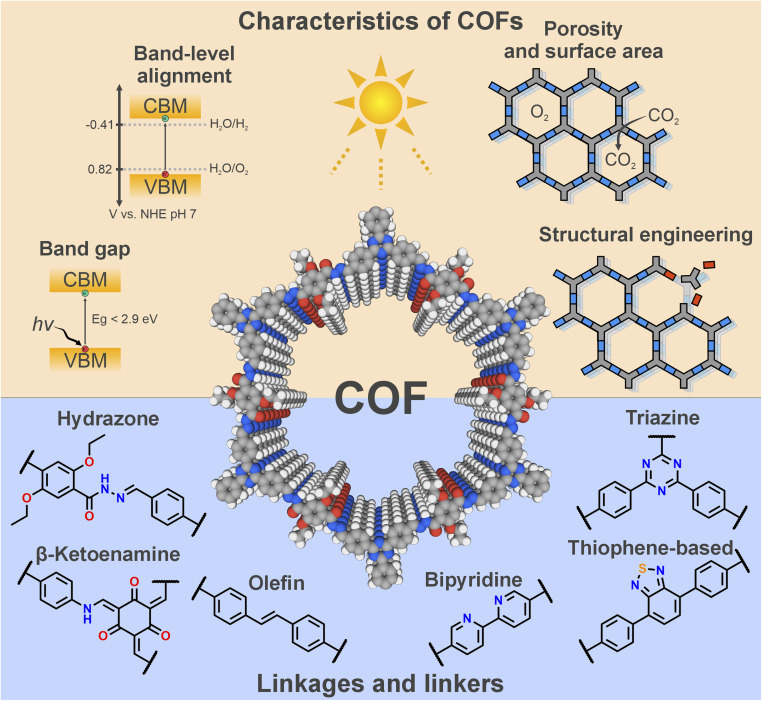
Schematic representation of some important characteristics of COFs as photocatalysts and some relevant linkages and linkers described in the literature.

Their covalent nature, semiconducting properties and intrinsic porosity endow COFs with a set of desirable properties that make them highly effective in catalytic applications, as illustrated in Fig. 2. Most organic linkers employed in COF synthesis are (hetero)aromatic systems, where the delocalization of π-electrons through the extended structure enhances electronic conjugation, facilitating photoexcitation with low-energy photons. Consequently, many COFs exhibit optical band gaps below 2.9 eV, enabling absorption of visible light (*λ* > 420 nm), a key prerequisite for solar energy harvesting. However, the magnitude of the band gap alone does not determine photocatalytic efficiency. The energetic alignment of the conduction band minimum (CBM) and valence band maximum (VBM) must also satisfy the thermodynamic requirements of the targeted redox reactions. In this regard, COFs typically possess sufficient reduction potential to drive reactions such as proton reduction, CO_2_ reduction, or N_2_ fixation. Nonetheless, the relatively shallow (*i.e.*, negative) VBM of COFs, a characteristic feature of electron-rich organic frameworks, can limit their performance in oxidation reactions such as the OER. However, it still provides sufficient oxidizing power to drive the oxidation of alcohols and thioethers. Moreover, the incorporation of transition metals such as Ru or Co can help overcome kinetic limitations, thereby improving the overall photocatalytic performance.^15,16^

Beyond their intriguing electronic properties, COFs possess an inherently reticular architecture that imparts them with a range of characteristics highly advantageous for catalysis. On the one hand, the well-defined porosity and high surface area of COFs play a critical role in photocatalytic processes. These features facilitate efficient diffusion of reactants through the framework, ensure high accessibility and utilisation of active sites, and allow reactions to occur within confined pores, where the local microenvironment—including local pH or concentration of intermediates—differs significantly from that of the bulk solution. Furthermore, the intimate contact between reactants and active sites promoted by the porous structure helps to suppress charge recombination, as the distance travelled by charge carriers before collection is reduced, enhancing the overall redox efficiency. On the other hand, the modular and designable nature of COFs allows precise structural engineering, enabling control over key parameters such as morphology, topology, crystallinity, and functionality. In this context, the incorporation of multiple functionalities within a single framework—yielding multivariate COFs—represents a powerful strategy to expand structural diversity and achieve synergistic combinations of properties. This concept can be further extended to mixed-length multivariate COFs, where linkers of mismatched lengths generate well-ordered yet compositionally complex architectures.^17^ Rational incorporation of diverse functionalities into COFs can greatly enhance their photophysical performance—for example, by improving charge separation in donor–acceptor architectures, or by tailoring light absorption through the inclusion of specific photoactive units adjacent to catalytic sites.^18^

Taking advantage of the characteristics mentioned above, a wide range of COFs with different chemical functionalities has been reported as efficient photocatalysts. Here we highlight a few prototypical linkages and linkers that keep reoccurring throughout the COF photocatalysis literature (Fig. 2): The incorporation of heteroatoms such as nitrogen and sulphur has proven particularly beneficial for photocatalysis. For instance, triazine moieties and thiophene-based units are known to promote charge-carrier transport and enhance local electronic conductivity, both of which are essential for efficient light harvesting.^19,20^ Furthermore, linkers such as bipyridine can coordinate transition metals, which not only act as active sites that activate substrates and accelerate reaction kinetics, but also broaden light absorption through metal-to-ligand (MLCT) or ligand-to-metal charge transfer (LMCT) transitions. In addition, nitrogen-rich motifs—such as bipyridine and diazine—can themselves serve as catalytic active sites, as observed in the ORR for H_2_O_2_ production.^21,22^

Besides the linkers, the covalent linkage connecting them represents another crucial component of a COF. It is a key structural element whose reversibility governs the well-known COF trilemma—the competition between stability, crystallinity, and functionality inherent to COFs.^23^ Moreover, the electronic nature of the linkage strongly influences the extent of π-electron delocalization across the framework. For example, the hydrazone linkage—featured in the seminal *Chem. Sci.* report—has been shown to facilitate charge separation and charge mobility under light irradiation.^24,25^ The stability of the framework during catalysis is also critically dependent on the robustness of the linkage, which often represents the weakest point susceptible to cleavage by hydrolysis. In this regard, β-ketoenamine and olefin linkages stand out due to their irreversible and highly resilient bonds. Despite their distinct electronic characteristics, both contribute beneficially to photocatalytic performance in different ways. The β-ketoenamine linkage enhances pore wettability, promoting reactions that involve proton transfer, and enables spatial separation of frontier orbitals, a feature ideal for donor–acceptor architectures. In contrast, olefin linkages extend π-conjugation throughout the framework, thereby improving charge-carrier mobility and reducing electron–hole recombination.

Undoubtedly, photocatalytic reactions are highly complex processes involving multiple interdependent factors. Parameters such as band gap, energy-level alignment, charge carrier mobility and separation, nature and accessibility of active sites, surface area, wettability, and dispersibility must all work in concert to achieve efficient performance. Owing to their extraordinary structural tunability, COFs allow many of these parameters to be systematically optimized according to the target reaction. This intrinsic versatility establishes COFs as a particularly intriguing class of photocatalysts.

### A look towards the future

Although significant progress has been achieved over the past decade, further understanding, improved processability, and enhanced scalability are still required to fully unlock the potential of COFs for solar fuel production and related applications. Advancing the catalytic efficiency of COFs must remain a priority in order to overcome intrinsic challenges such as high exciton binding energies and rapid exciton recombination processes typically observed in organic materials. Additionally, the implementation of emerging technologies—such as AI-assisted and automated synthesis and characterization—could greatly accelerate the discovery of new COFs, the screening of their photocatalytic properties, and, importantly, the establishment of robust and reproducible synthesis and catalytic testing protocols.

Furthermore, scaling COF photocatalysis to industrial levels remains a major challenge. Further developments in processability and scalability are needed to enable their integration into particle-based reactors, thin-film architectures, flat-panel configurations, and continuous-flow systems suitable for large-scale production. While structural diversity and complexity provide enormous value to COFs, the multistep synthesis of complex linkers and the reliance on non-abundant precursors hinder both scalability and economic viability, limiting the immediate industrial interest in COF-based systems for bulk commodities such as hydrogen production. Nevertheless, this scenario presents an opportunity for innovation. The same scientific enthusiasm and creativity that have propelled COF research over the past decade can drive new breakthroughs to overcome these barriers. In parallel, applications targeting fine or high-value chemicals through photoredox catalysis—where production costs are less constrained by commodity pricing—may offer a realistic and sustainable path for large-scale implementation of COF photocatalysts. Such directions could have transformative implications in fields such as pharmaceutical synthesis and agrochemical production.

As discussed throughout this commentary, a remarkable journey has unfolded since our first report in *Chem. Sci.* in 2014. Enormous progress has been achieved in understanding COFs as photocatalysts—both in terms of catalytic mechanisms and rational material design—thanks to researchers across different communities joining forces to pursue a common goal. This progress highlights the creativity of the scientific community and illustrates how curiosity continues to drive the creation of innovative functional materials. Most excitingly, despite the impressive advances made so far, intriguing challenges still lie on the horizon, promising a vibrant future for COF-based photocatalysis.

## Author contributions

Andrés Rodríguez-Camargo and Bettina V. Lorsch wrote the manuscript.

## Conflicts of interest

There are no conflicts to declare.

## Data Availability

There is no additional data associated with this article.
